# The quality of primary care performance in private sector facilities in Nairobi, Kenya: a cross-sectional descriptive survey

**DOI:** 10.1186/s12875-022-01700-3

**Published:** 2022-05-18

**Authors:** Gulnaz Mohamoud, Robert Mash

**Affiliations:** 1 Department of Family Medicine, Aga Khan University Hospital, Nairobi, Kenya; 2 Division of Family Medicine and Primary Care, Stellenbosch University, Cape Town, South Africa

**Keywords:** Primary health care, Primary care, Performance, Quality, Service delivery, Accessibility, Continuity, Coordination, Comprehensiveness

## Abstract

**Background:**

Integrated health services with an emphasis on primary care are needed for effective primary health care and achievement of universal health coverage. The key elements of high quality primary care are first-contact access, continuity, comprehensiveness, coordination, and person-centredness. In Kenya, there is paucity of information on the performance of these key elements and such information is needed to improve service delivery. Therefore, the study aimed to evaluate the quality of primary care performance in private sector facilities in Nairobi, Kenya.

**Methods:**

A cross-sectional descriptive study using an adapted Primary Care Assessment Tool for the Kenyan context and surveyed 412 systematically sampled primary care users, from 13 PC clinics. Data were analysed to measure 11 domains of primary care performance and two aggregated primary care scores using the Statistical Package for Social Sciences.

**Results:**

Mean primary care score was 2.64 (SD=0.23) and the mean expanded primary care score was 2.68 (SD=0.19), implying an overall low performance. The domains of first contact-utilisation, coordination (information system), family-centredness and cultural competence had mean scores of >3.0 (acceptable to good performance). The domains of first contact-access, coordination, comprehensiveness (provided and available), ongoing care and community-orientation had mean scores of < 3.0 (poor performance). Older respondents (*p*=0.05) and those with higher affiliation to the clinics (*p*=0.01) were more likely to rate primary care as acceptable to good.

**Conclusion:**

These primary care clinics in Nairobi showed gaps in performance. Performance was rated as acceptable-to-good for first-contact utilisation, the information systems, family-centredness and cultural competence. However, patients rated low performance related to first-contact access, ongoing care, coordination of care, comprehensiveness of services, community orientation and availability of a complete primary health care team. Performance could be improved by deploying family physicians, increasing the scope of practice to become more comprehensive, incentivising use of these PC clinics rather than the tertiary hospital, improving access after-hours and marketing the use of the clinics to the practice population.

## Introduction

The World Health Organization (WHO) defines primary health care (PHC) as ”a whole-of-society approach to health that aims to maximise the level and distribution of health and well-being” and regards PHC as the foundation of any health care system [1, 2, 3]. The Astana Declaration, signed in 2018, emphasised the need for governments to commit to achieve PHC services that are integrated, cost-effective, available, accessible, comprehensive and of high quality [4, 5]. The World Health Assembly (2019) also acknowledged the important role of providing PHC in order to achieve universal health coverage (UHC) through accessible health care that is of high quality [6]. However, due to weaknesses in PHC systems, such as fragmented care, insufficient funding, scarce human resources and poor quality of care, especially in low-and middle-income countries (LMICs), many countries have not yet delivered on these commitments [6]. Additional challenges have been noticed in recent years, such as the coronavirus pandemic, increases in prevalence of non-communicable chronic diseases and the impact of climate change [7]. However, countries with well-functioning PHC systems have better health outcomes, better equity, as well as more efficient, responsive and resilient health systems [8, 9, 10].

The WHO has identified three levers to improve PHC: multi-sectoral policy and action, empowered people and communities, and integrated health care services with emphasis on primary care and essential functions of public health [3]. Primary care (PC) is defined as a “key process in the health system that supports first-contact, accessible, continued, comprehensive and coordinated patient-focused care” and acts as a gatekeeper to other levels of care [3, 11].

Primary care in sub-Saharan Africa (SSA) faces difficulties such as hospital-centred priorities, health care fragmentation by vertical programmes, resource limitations, the burdens of communicable and non-communicable diseases, and reliance on low level and sometimes inadequately trained health care providers [1, 12, 13]. Primary care is the main point of entry for most people seeking health care, and yet PC in SSA lacks the ability to provide high quality care [12, 14]. The key elements of high quality service delivery in PC are: easy access for people with health problems, continuity, comprehensiveness, coordination, and person-centredness [8].

Achieving the goal of UHC also requires evaluating the quality of PC and improving the key elements of PC [15]. The need to measure these key elements is highlighted by the Primary Health Care Performance Initiative and in the Primary Care Assessment Tool [16, 17]. In SSA, a gap exists in the measurement of PC performance [9], and the absence of such information impedes the ability of policymakers and implementers to identify areas that need improvement as well as prioritise the use of resources [8].

In Kenya, numerous efforts have been made to achieve UHC by increasing access to and utilisation of PHC, through the introduction of free services as well as health insurance [18, 19]. Despite the increase in utilisation and broader coverage of the population, the measurement and quality of PC services remains challenging [18, 19]. In Kenya, PC is delivered by nurses, clinical officers (mid-level practitioners) and doctors, supported by other health care workers [20]. However, nurses are the main providers of primary care in the public sector and community health volunteers also offer PC in some regions/counties [20]. General practitioners (a doctor who has studied for a medical degree and passed their internship) offer services mostly in the private sector, although the majority do not have postgraduate training [21]. Specialist training in family medicine is available [22, 23], but the number of family physicians in Kenya is very limited [24].

The private health care system in Kenya provides 52% of all health care services and may have a bigger role to play in the future [18, 20]. Due to diversity and fragmentation of the private PC system, there is little data on the strengths and weaknesses of key elements of PC service delivery [8, 18]. Private sector PC is also varied and diverse in terms of geographical location, types of practice and organisation, which makes measurement of quality complex and difficult [25].

Therefore, the study aimed to evaluate the quality of PC performance in private sector facilities in Nairobi, Kenya. The objective was to asses the users’ experience of PC in terms of accessibility, comprehensiveness, continuity, coordination, community-orientation, primary health care team as well as aspects of person-centredness. Gaps in desired performance could be identified to inform tailored interventions for improvement.

## Methods

### Study design

This was a cross-sectional descriptive survey of patients in primary care using the Kenyan Primary Care Assessment Tool (KE-PCAT).

### Setting

This study was carried out in 13 primary care clinics within the city of Nairobi, run by the general practitioners (GPs). All the clinics were operated by a private health care organisation, affiliated with a private tertiary care referral hospital. These were ambulatory primary care clinics, offering services to all age groups in urban, semi-urban and peri-urban areas of Nairobi. The clinic staff included receptionists, registered nurses, laboratory technicians, radiographers and pharmacy technicians. All patient records were captured in the electronic medical record system at the clinics associated with this organisation and accessed only by the medical personnel working at these clinics. The clinics provided promotive, preventative and curative services for all age groups. The clinics had a dispensing pharmacy, laboratory and offered referral services to the specialists’ clinics (including family medicine) at the tertiary hospital. The patients came from diverse socio-economic backgrounds and most had private medical insurance by virtue of their employment. Previous studies at the same clinics showed that most of the patients spoke English and were well educated [26, 27].

### Study population and sample size

The study population included patients aged 18 years and above at the 13 primary care clinics. These patients should have attended the same clinic at least three times prior as they were required to have experienced the care provided [28]. Those who did not provide consent or the number of visits were less than three were excluded from the study. All patients below 18-years of age were also excluded.

These primary care clinics served approximately 15275 patients on a monthly basis. Therefore, the sample size calculation was based on a population of 20,000 patients, since calculations for the sample size do not change markedly in populations over 20,000. The calculation was based on an expected proportion of 61% of users having a good primary care score (score >3) [13], a 5% margin of error and 95% confidence interval. Sample size was calculated using Fischer’s formula that gave a figure of 375, and after adjusting for 10% of incomplete responses, the minimum sample size required was 412.

### Sampling strategy

The sample size of 412 was distributed amongst the 13 clinics proportional to the monthly workload. Patients that met the inclusion criteria were systematically sampled at each clinic until the sample size was achieved. If the patient did not provide consent, the next consenting patient was selected as per the systematic approach to sampling.

### Data collection tool

The Primary Care Assessment Tool (PCAT) was originally developed at the Johns Hopkins Populations Care Policy Centre for underserved populations in USA [17, 29]. It was cross-culturally validated and first adapted for the African context in South Africa [28, 29].

The PCAT enables an evaluation of PC performance in terms of access, comprehensiveness, continuity, coordination, community orientation, family-centredness, cultural competence and the primary health care team [9, 28].

The short user’s version of the South African PCAT (ZA-PCAT) was validated and adapted for the Kenyan PC context (KE-PCAT). The heads of the Departments of Family Medicine of the five academic institutions in Kenya and their senior faculty, who understood the key principles of PC and the Kenyan context, participated in the validation process. The content of the tool was reviewed by the panel that also included the principal investigator. The reviewers ensured that the questions were relevant and appropriate for the Kenyan context while preserving the integrity of the tool.

The panel achieved consensus (>70% of panel) on the content of the domains and items of the PCAT. From an original of 97 questions, two items were excluded as they were not relevant to the Kenyan context. Items requiring rephrasing for the local context were identified and the demographic section was adapted, taking into consideration the local socio-economic conditions and terms.

The revised tool was then assessed for feasibility and understanding through a pilot study, carried out at a PC clinic belonging to the same organisation, outside the Nairobi County, that was not a part of the study. There was no change made to the KE-PCAT after the pilot study.

The final version of the KE-PCAT tool comprised of 11 domains (Table 1); first contact (access), first contact (utilisation), ongoing care, coordination (system), coordination (information), comprehensiveness (services available), comprehensiveness (services provided), family-centredness, community orientation, cultural competence and the primary health care team. In addition, data on the extent of affiliation to the PC clinics, self-reported health assessments and socio-demographic information were collected. Most items were measured using a 4-point Likert scale from 1 (definitely not), 2 (probably not), 3 (probably) and 4 (definitely). There was also the option 'not sure, or don't remember'.

**Table 1:** Domains, items and definitions of the PCAT.

Domains	Number of items	Definition
1. First contact (access)	5	The provision of primary care services that are accessible when a need for care arises. First contact refers to the primary care provider being responsible for assisting the client to enter the healthcare system for each non-referred provision of health care.
2. First contact (utilisation)	3	The utilisation of primary care services when a need for care arises. First contact refers to the primary care provider being responsible for assisting the client to enter the healthcare system for each non-referred provision of health care.
3. Ongoing care	9	The use of a regular source of care over time that is not limited to certain types of healthcare needs. Longitudinally involves the development of a patient–provider relationship based on established trust and a knowledge of the patient and his/her family. A ‘health care home’ is thus established for each patient to promote the provision of ongoing care regardless of the presence or absence of disease.
4. Coordination (system)	10	Linking of healthcare events and services. Primary care has the responsibility and obligation to transfer information to and receive it from other resources that may be involved in the care of a client, and to develop and implement an appropriate plan for healthcare management and disease prevention.
5. Coordination (information)	3	Coordination requires the establishment of mechanisms to communicate information and the incorporation of that information into the client’s plan of care.
6. Comprehensiveness (available)	21	Primary care makes available a range of essential personal health services that promote and preserve health and provide care for illness and disability.
7. Comprehensiveness (provided)	9	Primary care offers a range of essential personal health services that promote and preserve health and provide care for illness and disability.
8. Family-centredness	3	Care understands the impact of family characteristics on the genesis and prevention of ill health, as well as the response to both medical and psychosocial interventions. Family-centred primary care recognises and incorporates knowledge of the family context (resources, risk factors, social factors) into the planning and delivery of primary care.
9. Community orientation	6	Care refers to efforts to recognise the primary care needs of a defined population. The effective delivery of services to individuals and communities is based on an understanding of community needs and the integration of a population perspective in the provision of health care. Primary care providers contribute to and participate in community assessment, health surveillance, monitoring, and evaluation.
10. Culturally competent	5	Care incorporates cultural references into the provision of primary care. Services are designed to be acceptable to people in the community, who may be distinguished by common values, language, heritage, and beliefs about health and disease. The views of these groups should be determined and incorporated into decisions involving policies, priorities, and plans related to the delivery of healthcare services.
11. PHC team available	6	The availability of members of the multidisciplinary primary health care team such as social workers, therapists or community health workers.
12. Primary care score	(Total)	Mean of the scores for: first contact (utilization); first contact (access); extent of affiliation with a place/doctor; ongoing care; coordination; coordination (information); comprehensiveness (services available); comprehensiveness (services provided).

Source: Evaluating the performance of South African primary care: a cross-sectional descriptive survey [13]

### Data collection process

After the patients were registered at the reception and prior to the triage process, the research assistant approached every third patient from the register. Those that consented, were asked about the number of times they had visited this facility. The participants who met the inclusion criteria were briefed about the study and those who agreed to participate were requested to give written consent. Research assistants administered the questionnaire in a private room. The interviews were conducted in English and minor clarification was provided where needed in Kiswahili. Research assistants were trained according to the PCAT training manual and were fluent in both English and Kiswahili. Data quality was checked by the principal investigator at the clinic, before entering into MS Excel for further analysis.

### Data analysis

Performance of the data analysis was according to the PCAT manual. The data were analysed by the first author using the Statistical Package for Social Sciences (SPSS) version 25.

A mean score was calculated for each domain from the associated items using the Likert scale from 1-4. Some items were reverse scored prior to the calculation and the options for ‘not sure or don’t remember’ were scored as per the PCAT manual.

In addition, a binary variable was constructed, where a mean score ≥ 3 was seen as 'acceptable to good performance' and < 3 as 'poor performance'. This threshold was recommended when the ZA-PCAT was validated [29].

To calculate affiliation with the PC clinics, users were first asked about the usual place or person where they sought care. They were then asked to identify any alternative place or person that they regularly visited and which place knew them best. The user’s extent of affiliation with the PC clinics was categorised into “high” for those who only attended the PC clinic in the study, “moderate” for those users that sometimes attended another place, but were known best at the study site, and “low” for users that sometimes attended another place and were also known best at the alternative place.

The PC score was calculated as the mean of the domain scores for affiliation, first contact (utilisation), first contact (access), ongoing care, comprehensiveness (services available), and comprehensiveness (services provided). The expanded PC score also included the domains of family-centredness, community orientation, cultural competence and the primary health care team.

Continuous variables were summarised using means and standard deviations (SD) or medians and inter-quartile ranges (IQR), depending on the distribution of the data. Categorical data was summarised using frequency counts with the corresponding percentages. Chi-square test compared the domains and socio-demographic variables with the PC score, when the data was categorical. When necessary post hoc analysis of the chi square test was performed.

## Results

The KE-PCAT was administered to 412 participants (Table 2). The majority were female (55.1%) and the median age of the users was 34.0 (IQR: 28.0-42.0). Most of the participants were in full time employment (58.7%), university graduates (73.5%) and living in permanent dwellings (99.3%). The users’ extent of affiliation with the PC facility was seen as “high” in 249 (60.4%), “moderate” in 95 (23.1%) and “low” in 65 (15.8%).

**Table 2:** User characteristics (*N*=412)

Variables	N	%
**Gender**		
Male	185	44.9
Female	227	55.1
**Age group (years)**		
20-29	107	26.0
30-39	176	42.7
40-49	86	20.9
50-59	37	9.0
60-69	6	1.5
**Preferred language**		
English	219	53.2
Kiswahili	186	45.1
Others	6	1.5
Refuse to answer	1	0.2
**Employment**		
Employed-full time	242	58.7
Employed-part time	59	14.3
Self-employed (informal sector)	28	6.8
Self-employed (formal sector)	17	4.1
Student	24	5.8
Homemaker	20	4.9
Retired/pensioner	20	4.9
Disabled	1	0.2
Refuse to answer	1	0.2
**Education level**		
Only primary	10	2.4
Only secondary	22	5.3
College	68	16.5
University	303	73.5
Other	9	2.2
**Water**		
Piped water (compound)	407	98.8
Piped water (yard)	2	0.5
Piped water (nearby)	4	1.0
**Electricity**		
Yes	409	99.3
Refuse to answer	3	0.7
**Type of dwelling**		
Permanent	409	99.3
Refuse to answer	3	0.7
**Toilet**		
Yes	410	99.5
No	2	0.5
**Self-reported health status**		
Excellent	10	2.4
Very good	74	18.0
Good	171	41.5
Fair	137	33.3
Poor	20	4.9
**Chronic condition**		
Yes	45	10.9
No	367	89.1

**Fig. 1:**
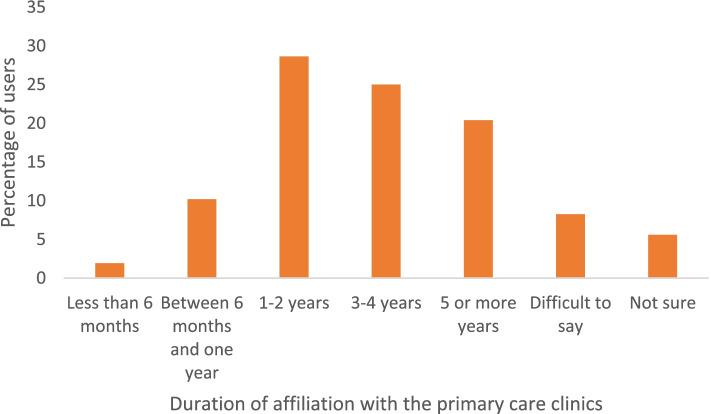
Users’ affiliation with the primary care clinics.

Figure 1 shows the duration of affiliation with the PC facilities. The majority of the participants had been affiliated for 1-4 years (53.4%). The median number of times that the users attended the clinic in the last 2-years was 4.0 (IQR: 3.0-6.0).

**Fig. 2:**
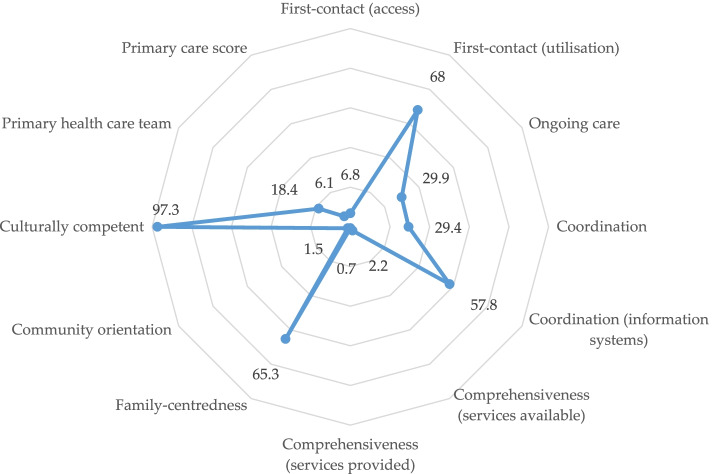
Proportion of respondents evaluating each domain as acceptable to good

Table 3 shows the performance scores for each domain. The mean PC score was 2.64 (SD=0.23) and the mean expanded PC score was 2.68 (SD=0.19), implying an overall low performance. The domains of first contact (utilisation), coordination (information), family-centredness and cultural competence had mean scores of 3.0 or more, suggesting an acceptable to good performance. All other domains had a mean score of less than 3.0, suggesting a poor performance. The proportion of respondents giving an acceptable or good PC score for each domain is also shown in a radar chart in Fig. 2.

**Table 3:** Performance scores for KE-PCAT domains (*N*=412)

Domains	Performance scores			
	Mean	SD	Score < 3 n (%)	Score >3 n (%)
First contact (utilisation)	3.1	0.6	132 (32.0)	280 (68.0)
First contact (access)	2.3	0.3	384 (93.2)	28 (6.8)
Ongoing care	2.8	0.3	289 (70.1)	123 (29.9)
Coordination*	2.9	0.5	12 (70.6)	5 (29.4)
Coordination (information)	3.0	0.5	174 (42.2)	238 (57.8)
Comprehensiveness (services available)	2.1	0.3	403 (97.8)	9 (2.2)
Comprehensiveness (services provided)	2.1	0.3	409 (99.3)	3 (0.7)
Family-centredness	3.1	0.6	143 (34.7)	269 (65.3)
Community orientation	2.0	0.4	406 (98.5)	6 (1.5)
Culturally competent	3.7	0.4	11 (2.7)	401 (97.3)
Primary health care team	2.1	0.6	336 (81.6)	76 (18.4)
Total primary care score	2.6	0.2	387 (93.9)	25 (6.1)
Expanded primary care score	2.7	0.2	393 (95.4)	19 (4.6)

*N=17 only, representing the number of participants referred to a specialist or hospital service. This domain was excluded from the calculation of the PC scores as there were so few respondents

Table 4 shows the associations between the socio-demographic characteristics of the users and the PC score. A borderline significant association was found between age groups and the PC score (*p*=0.05). The post hoc analysis showed that the significance was due to a higher score amongst the 60-69 year olds (*p*=<0.0001), but all other age groups were not significantly different. There was also an association between higher affiliation with the clinic and a higher PC score (*p*= 0.01).

**Table 4:** Relationship between the socio-demographic characteristics and the primary care performance score (*N*=412).

Variables	Score <3 n (%)	Score **>** 3 n (%)	N	*p*-value
**Gender**				0.25
Male	171 (92.4)	14 (7.6)	185	
Female	216 (95.2)	11 (4.8)	227	
**Age Group**				0.05
20-29	99 (92.5)	8 (7.5)	107	
30-39	168 (95.5)	8 (4.5)	176	
40-49	82 (95.3)	4 (4.7)	86	
50-59	34 (91.9)	3 (8.1)	37	
60-69	4 (66.7)	2 (33.3)	6	
**Employment**				0.84
Employed-full time	229 (94.6)	13 (5.4)	242	
Employed-part time	54 (91.5)	5 (8.5)	59	
Self-employed (informal sector)	27 (96.4)	1 (3.6)	28	
Self-employed (formal sector)	16 (94.1)	1 (5.9)	17	
Student	23 (95.8)	1 (4.2)	24	
Homemaker	19 (95.0)	1 (5.0)	20	
Retired/pensioner	17 (85.0)	3 (15.0)	20	
Disabled	1 (100.0)	0 (0.0)	1	
Refuse to answer	1 (100.0)	0 (0.0)	1	
**Education**				0.88
Primary	10 (100.0)	0 (0.0)	10	
Secondary	21 (95.50	1 (4.5)	22	
College	64 (94.1)	4 (5.9)	68	
University	284 (93.7)	19 (6.3)	303	
Other	8 (88.9)	1 (11.1)	9	
**Users affiliation**				0.01
Low	65 (100.0)	0 (0.0)	65	
Moderate	93 (97.9)	2 (2.1)	95	
High	229 (92.0)	20 (8.0)	249	

## Discussion

In this private healthcare setting, the majority of the patients were young adults, female, employed, university graduates and resided in permanent dwellings. Most of them self-rated their health status as good and did not have chronic conditions.

Patients rated the clinics highly in terms of the information systems that helped to coordinate their care as well as in terms of the cultural competence and family-orientation of the GPs. On the other hand, they thought the clinics were not comprehensive in the range of services available and provided, and did not have a complete PHC team. There was little commitment to ongoing care, although patients also rarely had chronic conditions. Likewise, patients were rarely referred to the hospital and it was therefore difficult to assess coordination of care for such referrals. Despite high utilisation, the clinics were not always accessible at convenient times. The clinics did not have a community orientation as they tended to focus only on the patients that attended the facilities and did not have a well-defined geographic community or population at risk that they felt responsible for. Overall, the mean PC score and the mean expanded PC score implied a low performance.

The study showed a significant association between higher PC scores and older adults, although the patients were mostly young adults with good to excellent health and few chronic conditions. These findings were similar to another study carried out in the same clinics [27]. The low prevalence of chronic conditions could also be due to the perception, as reported in another study, that GPs were not able to deal with certain chronic conditions such as HIV, diabetes and mental illness, and that it was better to attend specialist care at the main hospital [26]. Despite the presence of chronic illness, the health status may still be reported as good, as shown in the study from South Africa [28]. Our study showed no relationship between self-rated health status and the PC score, although a study in Korea reported that a higher PC score was associated with a better self-rated health status [30].

First-contact access, which included the clinics’ operational processes such as opening hours, telephonic access and the provision of emergency services after hours, was rated poor. This rating could have been influenced by the COVID-19 pandemic, the county lock-down, and curfews leading to earlier closure of the clinics. In addition, telephonic consultations are not reimbursed by insurance companies in Kenya, unlike in high-income countries [27, 31]. A previous study carried out at these facilities showed high satisfaction with the clinics opening hours and waiting times, though concerns were expressed with the appointment system and easy access by phone to the GPs [27].

Similar findings for access scores were reported in Canada (mean score 2.2) [32], South Africa (mean score 2.5) [28], and Malawi (mean score 2.8) [33], showing that this aspect of care needs to be addressed in many PC systems. In addition, several studies carried out across Africa in the public sector, reported low levels of patient satisfaction with access to PC, either due to inconvenient opening times and appointments, staff shortages or lack of emergency services after hours [28, 34, 35, 36]. On the other hand, private clinics in Vietnam [37], Hong Kong [38], and China [39], showed greater accessibility, attributed to a stronger culture of customer service. Undoubtedly, difficulties in accessing PC can lead to inappropriate use of emergency services at the nearest hospital [13].

First-contact utilisation scored highly, showing that patients tended to use the clinics when they had a health issue or needed a check-up. Stronger affiliation was also associated with higher PC scores. Such high utilisation might be due to the physical proximity of the clinics [37], and satisfaction with the services offered [38], although such services were limited in scope [27].

Although utilisation and long term affiliation was reported as good, the score for relational continuity and ongoing care was poor. The young and generally healthy practice population needed acute episodic care more than chronic care and may therefore not have formed strong relationships with their GPs. Poor continuity, however, is usually associated with more fragmented care and opportunities that are missed for health promotion and disease prevention [40, 41].

Other studies in this practice population have shown low expectations of the clinic services and little preference for a specific GP, although high confidence was shown in the GPs ability to manage mostly minor acute problems in healthy young adults [26, 27]. Another reason for the gap in continuity, could be the lack of gate-keeping and availability of medical insurance cover, which allows patients to easily access the hospital specialists [27].

The GPs have also been shown to lack person-centred communication skills, which are important for building relationships, fostering continuity and ensuring patient satisfaction, which can also impact health outcomes [27, 42, 43]. In addition, relational continuity may not be part of normative health seeking expectations in the Kenyan context, although it is normative in other health systems [27, 44]. High utilisation of the facilities and a good electronic medical record system in this study did not translate into good continuity of care, which has been shown in studies conducted in South Africa [13, 36], Malawi [33], and Vietnam [37]. Improving ongoing care will be important if these clinics become more comprehensive and manage more chronic conditions.

The patients rated the coordination of information systems as good, which is most likely due to the efficient and integrated electronic medical record system. Thus, the availability and transfer of information to facilitate patient’s care could guide the development of an appropriate management plan [28, 40]. High scores in care coordination due to good record keeping was also found in a studies carried out in the public sector in South Africa and Vietnam [13, 37].

Users rated sequential coordination as barely acceptable, which indicated gaps in the transfer of information and care coordination between the PC facilities and the tertiary care hospital. This could be related to patient’s being non-compliant to follow-up, lack of coordination between the GPs and the specialists, and limited relational continuity. In addition, easy access to specialist services at the hospital, without the need for referral, could also contribute to a low commitment to sequential coordination [27]. In many primary care systems, gatekeeping is obligatory in order to improve the efficiency and equity of the system, thereby making the coordination of care essential by the PC provider [26, 28].

The provision of comprehensive services to meet the health needs of the community is a unique feature of PC in a generalist and undifferentiated environment. Comprehensiveness implies services across the whole burden of disease, the whole life course and from health promotion to palliation [28]. In our study, patients rated comprehensiveness as poor. Primary care in LMICs has historically been selective and driven by vertical disease-orientated programmes as shown by studies conducted in Malawi [33], South Africa [28], Kenya [26], Vietnam [37], and Brazil [45]. Even in high income countries such as Canada, comprehensive care is still an issue, despite having high relational continuity with providers [32]. In addition, the training of doctors in Kenya does not prepare them for comprehensive primary care, although additional training in family medicine may narrow this gap [46, 47, 48]. Comprehensive care plays a fundamental role in care continuity and when both are not delivered at an acceptable level it has implications for health outcomes [28, 49, 41].

The low score for comprehensiveness may be related to services not being available or patients being unaware of services that could be offered by the GPs [26]. For example, patients have reported reduced confidence in the ability of the GPs’ to manage and provide care related to screening for cervical cancer, antenatal care and end of life issues [26]. Services may not be provided by the GPs due to the availability of hospital specialists [50], which in turn results in the GPs becoming deskilled [51]. General practitioners may also lack certain skills to provide essential PC in specific areas of surgery, women’s health, ear, nose and throat, ophthalmology and orthopaedics, which may result in increased overall costs and hospital visits [48, 52].

Family-centredness is related to person-centred holistic PC and helps in understanding the patient’s context [53]. Family-centredness was scored as acceptable to good in this study. Several studies have related geographical proximity [37], family medical insurance cover [26, 27], duration of affiliation, and high utilisation of PC, with higher family-centredness [36, 54]. On the other hand, evaluation of consultations in the same settings showed that the GPs did not explore the family and social context in more than half of the consultations [48]. Patients clearly felt that GPs were open to considering family in the consultations, although this was not borne out by actual observation of the consultations [48].

Users rated community orientation as low and it is recognised that engagement in the community is not a strong point for the private sector [6]. The private sector generally focuses on the practice population, as individuals come for a service, as opposed to the public sector. In Kenya, particularly, the public sector has prioritised community orientation in PHC service delivery [19]. Despite the facilities being located in different communities throughout Nairobi, the organisation did not have a vision for community engagement and health surveillance [28].

Users rated cultural competence the highest, which implies that GPs were competent at handling the diversity of languages, contexts, health beliefs and values during their consultations [55]. This could be attributed to the GPs and other staff respecting the legitimacy of different cultures or because GPs actually shared the same language and cultural background as the patients [28, 36, 53]. The need for cultural sensitivity in PHC was also highlighted in a study in Botswana [56].

The users rated the composition of members of the primary health care team as low, which could be due to lack of awareness of the available services [26], or gaps in access to a multidisciplinary team and comprehensive care [26, 27]. Despite the gap in the PC team, there was a high level of care coordination within the teams at the facilities [48]. Many of the disciplines usually found in PC were actually located in the tertiary hospital, such as family medicine, social work, physiotherapy, dentistry and dietetics [26].

### Strengths and limitations

This is a first-of-its-kind study to be carried out in the Kenyan private sector. The users’ recall of their past experiences during health care visits may have created a recall bias, although research assistants were able to clarify and explore the answers to questions during the interviews. The possibility of an obsequiousness bias was also reduced by the use of unknown research assistants, assurance of anonymity and independence from the provision of care at the facility. The results cannot be generalised outside of the organisation as all participants were recruited from a single-organisation and model of care. However, the findings might be similar in other private sector services that are organised along similar lines.

### Recommendations

An improvement in the availability of routine services on weekends and after-office hours would add value to the already existing high user’ utilisation with the facilities. The comprehensiveness of services and PC team need to be improved and marketed to the practice population, which should also improve continuity and coordination of care. Furthermore, creating awareness of the care package, incentivising use of PC rather than the tertiary hospital, and continuing professional development for GPs, could help in addressing the comprehensiveness of PC [57]. Thus, services can be offered more cost-effectively and conveniently in the PC clinics as opposed to the tertiary hospital. Deploying family physicians in these clinics, would contribute towards providing person-centred, continuous, coordinated and comprehensive care.

Consideration should be given to more community-orientated PC programs. This was a private sector organisation that was founded on a non-profit and philanthropic model that might be amenable to such a focus. This might also be achieved through public-private partnerships [28].

The success of interventions to improve the domains that scored poorly can be monitored and evaluated by further evaluations using the PCAT in continuous quality improvement cycles [28].

## Conclusion

These PC clinics in Nairobi showed gaps in their performance. Performance was rated as acceptable-to-good in first-contact utilisation, the information systems, family-centredness and cultural competence. However, patients gave low ratings in the performance related to first-contact access, ongoing care, coordination of care, comprehensiveness of services, community orientation and availability of a complete primary health care team. The PC score could be improved by deploying family physicians to the clinics, training of the GPs, increasing the scope of practice to become more comprehensive, incentivising use of PC rather than the tertiary hospital, improving access after-hours and marketing the use of the clinics to the practice population.

## Data Availability

The authors confirm that the data supporting the findings of this study are available on reasonable request to the authors.

## Abbreviations

AKUH: Aga Khan University Hospital
COVID-19: Coronavirus Disease 2019
GPs: General Practitioners
IQR: Inter quantile Range
KE-PCAT: Kenya-Primary Care Assessment Tool
LMICs: Low and Middle Income Countries
NACOSTI: National Commission for Science, Technology and Innovation
PC: Primary Care
PCAT: Primary Care Assessment Tool
PHC: Primary Health Care
SD: Standard Deviation
SPSS: Statistical Package for Social Science
SSA: Sub-Saharan Africa
UHC: Universal Health Coverage
WHO: World Health Organization
ZA PCAT: South African Primary Care Assessment Tool
